# Predictive model for BNT162b2 vaccine response in cancer patients based on blood cytokines and growth factors

**DOI:** 10.3389/fimmu.2022.1062136

**Published:** 2022-12-22

**Authors:** Angelina Konnova, Fien H. R. De Winter, Akshita Gupta, Lise Verbruggen, An Hotterbeekx, Matilda Berkell, Laure-Anne Teuwen, Greetje Vanhoutte, Bart Peeters, Silke Raats, Isolde Van der Massen, Sven De Keersmaecker, Yana Debie, Manon Huizing, Pieter Pannus, Kristof Y. Neven, Kevin K. Ariën, Geert A. Martens, Marc Van Den Bulcke, Ella Roelant, Isabelle Desombere, Sébastien Anguille, Zwi Berneman, Maria E. Goossens, Herman Goossens, Surbhi Malhotra-Kumar, Evelina Tacconelli, Timon Vandamme, Marc Peeters, Peter van Dam, Samir Kumar-Singh

**Affiliations:** ^1^ Molecular Pathology Group, Laboratory of Cell Biology & Histology, Faculty of Medicine and Health Sciences, University of Antwerp, Wilrijk, Belgium; ^2^ Laboratory of Medical Microbiology, Vaccine and Infectious disease Institute, University of Antwerp, Wilrijk, Belgium; ^3^ Multidisciplinary Oncological Center Antwerp (MOCA), Antwerp University Hospital, Edegem, Belgium; ^4^ Center for Oncological Research (CORE), Integrated Personalized and Precision Oncology Network (IPPON), University of Antwerp, Wilrijk, Belgium; ^5^ Department of Laboratory Medicine, Antwerp University Hospital, Edegem, Belgium; ^6^ Biobank, Antwerp University Hospital, Edegem, Belgium; ^7^ Scientific Directorate Epidemiology and Public Health, Sciensano, Brussels, Belgium; ^8^ Centre for Environmental Sciences, Hasselt University, Hasselt, Belgium; ^9^ Federal Public Service (FPS) Health, Food Chain Safety and Environment, Brussels, Belgium; ^10^ Virology Unit, Institute of Tropical Medicine Antwerp, Antwerp, Belgium; ^11^ Department of Biomedical Sciences, University of Antwerp, Edegem, Belgium; ^12^ Department of Laboratory Medicine, AZ Delta General Hospital, Roeselare, Belgium; ^13^ Clinical Trial Center (CTC), Clinical Research Centre (CRC) Antwerp, Antwerp University Hospital, University of Antwerp, Edegem, Belgium; ^14^ StatUa, Center for Statistics, University of Antwerp, Antwerp, Belgium; ^15^ Service Immune response, Scientific Directorate Infectious Diseases in Humans, Sciensano, Brussels, Belgium; ^16^ Scientific Directorate Infectious Diseases in Humans, Sciensano, Brussels, Belgium; ^17^ Division of Infectious Diseases, Department of Diagnostics and Public Health, University of Verona, Verona, Italy

**Keywords:** COVID-19 vaccine, BNT162b2, SARS-CoV-2, solid cancers, haematological malignancies, cytokines, chemokines, growth factors

## Abstract

**Background:**

Patients with cancer, especially hematological cancer, are at increased risk for breakthrough COVID-19 infection. So far, a predictive biomarker that can assess compromised vaccine-induced anti-SARS-CoV-2 immunity in cancer patients has not been proposed.

**Methods:**

We employed machine learning approaches to identify a biomarker signature based on blood cytokines, chemokines, and immune- and non-immune-related growth factors linked to vaccine immunogenicity in 199 cancer patients receiving the BNT162b2 vaccine.

**Results:**

C-reactive protein (general marker of inflammation), interleukin (IL)-15 (a pro-inflammatory cytokine), IL-18 (interferon-gamma inducing factor), and placental growth factor (an angiogenic cytokine) correctly classified patients with a diminished vaccine response assessed at day 49 with >80% accuracy. Amongst these, CRP showed the highest predictive value for poor response to vaccine administration. Importantly, this unique signature of vaccine response was present at different studied timepoints both before and after vaccination and was not majorly affected by different anti-cancer treatments.

**Conclusion:**

We propose a blood-based signature of cytokines and growth factors that can be employed in identifying cancer patients at persistent high risk of COVID-19 despite vaccination with BNT162b2. Our data also suggest that such a signature may reflect the inherent immunological constitution of some cancer patients who are refractive to immunotherapy.

## 1 Introduction

The field of vaccination against infectious disease has witnessed rapid advances of technology throughout the COVID-19 pandemic, including the development of various anti-SARS-CoV-2 vaccines, such as mRNA and vector vaccines. The BNT162b2 mRNA COVID-19 vaccine elicits a range of immunological responses, especially a strong anti-SARS-CoV-2 IgG response in healthy individuals, which starts waning after approximately 3-6 months (1–3). However, because the mechanisms determining the quality and quantity of immunological responses are not fully understood, this has led to concerns about the efficiency of these vaccines in immunosuppressed populations including patients with solid or hematological malignancies (4), especially when they are under active antineoplastic treatments. Several studies have shown that vaccine responses are compromised in patients with hematological malignancies under B cell depleting rituximab treatment, or with solid tumors receiving different chemotherapies (5–11).

Biomarkers of protection against SARS-CoV-2 infection generated by anti-SARS-CoV-2 vaccines have been studied for general population (12, 13). For instance, a post-vaccine anti-SARS-CoV-2 response with BNT162b2 in healthy volunteers is shown to be accompanied by alterations in systemic cytokine, chemokine, and specific growth factors (CCGs), including increase in interleukin (IL)-15, interferon gamma (IFN-γ), and IFN-γ-induced protein 10 (IP-10/CXCL10) after the primer vaccination dose, and by tumor necrosis factor alpha (TNF-α) and IL-6 after the booster vaccination dose (12). Importantly, transient increases in IL-15 and IFN-γ levels were also identified as biomarkers for anti-SARS-CoV-2 responses in a healthy population (12). However, none of these biomarkers are currently available for cancer patients, where such a marker can distinguish subgroups of patients which are poorly protected by SARS-CoV-2 vaccination and remain in need of additional (preventive) options (14–16). CCGs are not only important in the regulation of inflammation occurring in viral infections such as SARS-CoV-2 (12, 17–19) and influenza (20, 21), but also play an important role in the initiation and progression of cancers (22–25). We recently demonstrated significant alterations in levels of several CCGs in blood of cancer patients including, but not limited to, CCGs that play an important role in the adaptive immune response in antigen presentation and/or T-helper and B cell functions (25). In the present study, we propose a blood-based signature of cytokines/chemokines and growth factors that can be employed in identifying cancer patients at persistent high-risk of COVID-19 despite vaccination with BNT162b2.

## 2 Material and methods

### 2.1 Patient population and study design

A prospective, longitudinal, multi-cohort trial was initiated on February 15, 2021, in the Multidisciplinary Oncological Center Antwerp (MOCA), Antwerp University Hospital, Belgium, as described (5). Briefly, study participants aged 18 years or older with a life expectancy of at least six months were recruited. Pregnant or breastfeeding women and patients with an immunodeficiency unrelated to cancer treatment were not included. All study participants provided written informed consent. A total of 200 cancer patients recruited in this study received at least one dose of the BNT162b2 vaccine. One patient withdrew after the primer dose and was excluded from the study. CCGs before and after the primer dose were measured for 199 patients, including 158 patients with a solid tumor and 41 patients with a hematological malignancy (**Supplementary Table 1**). From these 199 patients, 187 patients received a booster dose 21 (± 2) days after the primer dose according to the study protocol. Nine patients received a delayed booster dose from 24 to 37 days due to an active SARS-CoV-2-CoV-2 infection or cancer treatment-related complications (5). The study was approved by the local ethics committee and was executed in accordance with Good Clinical Practice and the Declaration of Helsinki (ICH GCP E6(R2)). The regulatory sponsor was the Antwerp University Hospital (EudraCT number 2021-000300-38).

The included population with solid tumors mainly consisted of patients with breast malignancies (52.8%), followed by patients with gastroenterological (10.1%) and gynecological malignancies (10.1%). Among patients with hematological malignancies, 75.6% of patients had chronic lymphocytic leukemia or lymphomas and 19.5% patients had myeloid malignancies. On the basis of cancer and treatment modalities, we defined 4 cohorts: (i) patients with solid tumors (ST) receiving only chemotherapy (n = 63); (ii) ST patients receiving immunotherapy with or without chemotherapy (n = 16); (iii) ST patients receiving targeted or hormonal therapy (n = 79); and (iv) a combined group of hematological malignancy patients (n = 41) receiving either rituximab (n = 29), targeted therapy (n = 1), or an allogenic hematopoietic stem cell transplantation more than one year ago (n = 11).

### 2.2 Sample collection and processing

**Figure 1:**
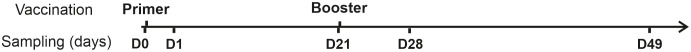
Timeline of the study. The BNT162b2 vaccine was administered on day 0 and 21. Heparin plasma samples for CCG analysis were collected on day 0 just prior to primer dose administration (D0), day 1 (D1), day 21 just prior to booster dose administration (D21), and day 28 (D28). For anti-RBD and anti-S1 serology, serum samples were collected on day 49 (D49) after the administration of the primer vaccine dose.

Plasma samples were taken at the day of study inclusion (day 0, just before administration of the primer dose), day 1 (the day after the primer dose), day 21 (just before administration of the booster dose), and day 28 (7 days after the booster dose). Serum samples were collected at day 49 (**Figure 1**). For detailed methods, refer to **Supplementary Information**.

### 2.3 Cytokine, chemokine and growth factor measurements in plasma

CCGs were measured in plasma samples on a multiplex platform (Meso Scale Discovery (MSD), MD, USA) using off-the-shelf (V-plex) and customized (U-plex) panels, according to the manufacturer’s instructions, as previously described (25). For detailed methods, refer to **Supplementary Information**.

In total, 36 CCGs relevant for SARS-CoV-2 infection or tumor growth and progression were measured. These constituted brain-derived neurotrophic factor (BDNF), basic fibroblast growth factor (bFGF), C-reactive protein (CRP), cutaneous T-cell attracting chemokine (CTACK), vascular endothelial growth factor receptor 1 (FlT-1), interferon β (IFN-β), interferon γ (IFN-γ), IL-1β, IL-1 receptor antagonist (IL-1Ra), IL-2, IL-4, IL-5, IL-6, IL-8, IL-10, IL-13, IL-15, IL-16, IL-17A, IL-18, IL-21, IL-33, IFN-γ induced protein 10 (IP-10; also called CXCL10), monocyte chemoattractant protein (MCP)-1, placental growth factor (PlGF), serum amyloid A (SAA), soluble intercellular adhesion molecule 1 (sICAM-1), soluble vascular cell adhesion molecule 1 (VCAM-1), active and total (acid activated) tumor growth factor β (TGF-β), angiopoietin receptor 1 (Tie-2), TNF-α, thymic stromal lymphopoietin (TSLP), vascular endothelial growth factor (VEGF)-A, VEGF-C and VEGF-D. An additional 5 CCGs were measured in a random subset of plasma samples from 100 cancer patients. These were granulocyte colony stimulating factor (G-CSF), granulocyte-macrophage colony-stimulating factor (GM-CSF), IL-7, IL-9, and macrophage inflammatory protein (MIP)-1α.

### 2.4 Anti-RBD IgG measurements in serum

Anti-RBD immunoglobulin G (IgG) levels were measured in serum samples with an enzyme-linked immunosorbent assay (ELISA) as previously described (5). A threshold for anti-RBD IgG of 200 IU/mL predicted a neutralization response required for 50% protection against symptomatic SARS-CoV-2 infection (99%-100% specificity at a sensitivity of 94.9%). As such, this threshold was used to differentiate high from low anti-SARS-CoV-2 serological responders, as described (5).

### 2.5 Statistics

Group differences in CCG profiles of patients belonging to different treatment cohorts were investigated by Partial Least-Squares Discriminant Analysis (PLS-DA) using MetaboAnalyst (version 5.0). For this, data was primarily normalized with autoscaling and log10 transformation as described (25). Different timepoints (before and after vaccination) were compared using a paired t-test on log-transformed data (SPSS v27). Good vs. poor anti-SARS-CoV-2 IgG responders and patients with vs. without severe adverse events were compared using a two-sample t-test on log-transformed data (SPSS v27). To evaluate correlation between quantitative IgG levels and CCG concentrations, a Spearman correlation coefficient was utilized (R, version 4.1.0, http://www.rstudio.com/). A *p*-value of < 0.05 (uncorrected) was considered statistically significant.

For the identification of the main predictors of qualitative response (good/poor responder), receiver operating characteristic (ROC) curves were constructed utilizing MetaboAnalyst. To further predict good/poor responder with a combined model of CCG levels, machine-learning-based Random Forest classifiers (RFC) were built (Python, package sklearn v2.0, ttp://scikit-learn.sourceforge.net). The main outcome variable was the development of an adequate immune response. To account for imbalanced groups, the Synthetic Minority Oversampling Technique (SMOTE, Python package imblearn 0.8.0) was utilized where 80% of the data was utilized as training set and the remaining 20% as test set. The models were bootstrapped 10 times and features for each model were selected based on 1) feature importance, 2) statistics from good vs. poor responder, 3) Individual ROC curve analysis, and, 4) a Pearson correlation matrix for independence of variables. Confusion matrices and ROC curves were drawn to calculate area under the curve (AUROC) value to verify reliability and to evaluate the performance of the constructed models.

## 3 Results

### 3.1 Activation of early immune responses by BNT162b2 in cancer patients

**Figure 2:**
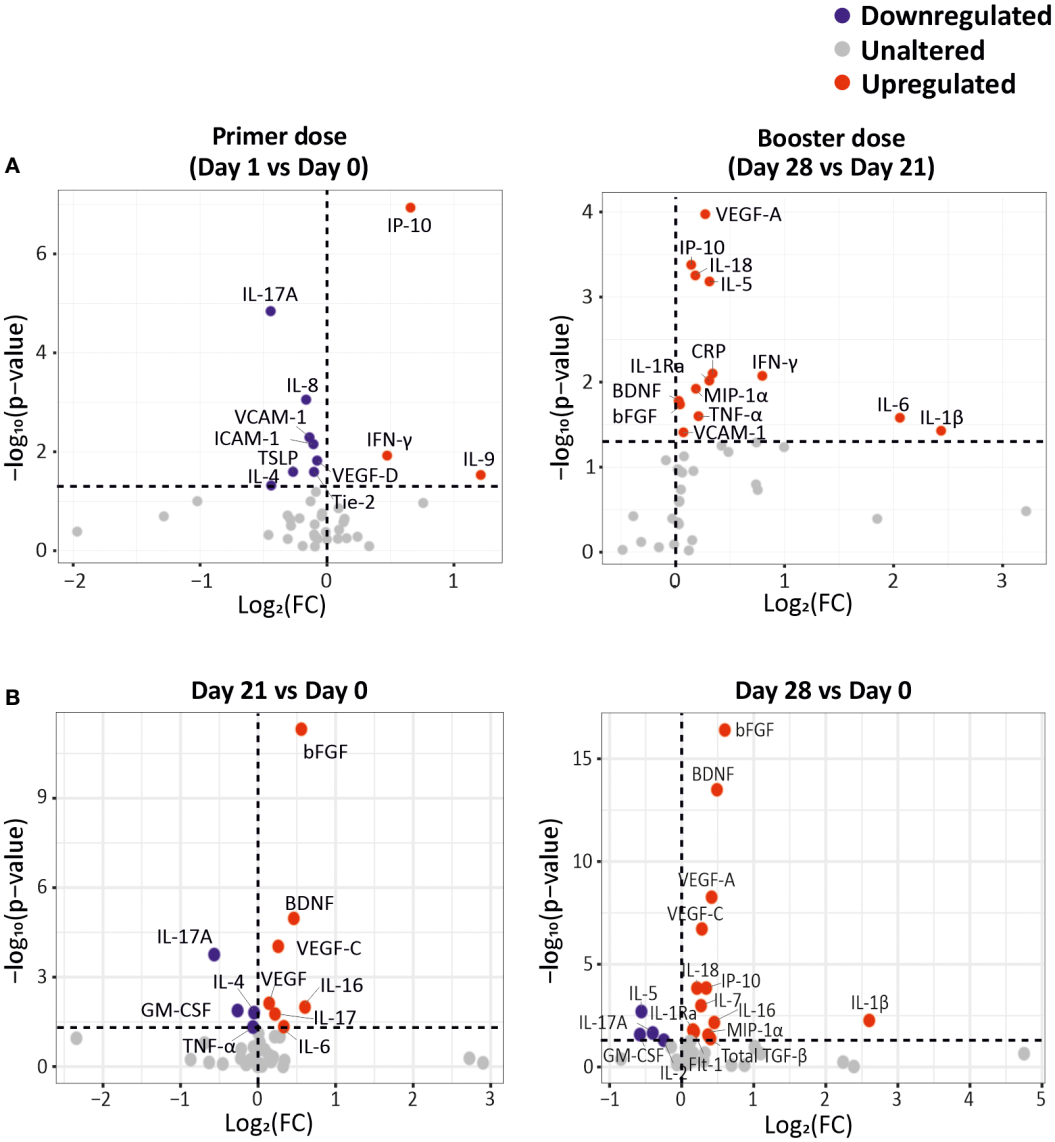
CCG alterations as a response to primer and booster dose vaccinations in cancer patients. **(A)** Differentially expressed CCGs after the administration of the primer and booster doses, compared to the CCG levels prior to vaccine administration. **(B)** Differentially expressed CCGs at day 21 and day 28, compared with baseline day 0. *P*-values were calculated using paired t-test. The vertical dotted line represents no change. The horizontal dotted line represents a *p*-value of 0.05.

We observed a significant alteration of 23 CCGs after administration of the primer and/or the booster dose of the BNT162b2 vaccine in cancer patients under active treatment (**Figure 1**). Specifically, a day after the administration of the primer dose (day 1 vs. baseline day 0), anti-viral responses such as IFN-γ and IP-10 as well as T cell growth factor IL-9 were significantly upregulated (**Figure 2A** **, left panel**), suggesting, as expected, the importance of these immune mediators in the initial immune response to the vaccine.

On the other hand, we measured a downregulation of several CCGs after administration of the primer dose, such as IL-17A, IL-8, IL-4, TSLP, VCAM-1, ICAM-1, Tie-2, and VEGF-D. Most of these analytes are crucially involved in the adaptive immune response or in cancer progression (26, 27). For example, downregulation of TSLP, that has an important role in the maturation of T cell populations and in enhancing Th2 responses (28), and of IL-4, a key Th2 cytokine with profound effects on B cell function, could be detrimental to the development of an adaptive immune response in the studied cancer patients (**Figure 2A**, left panel).

Seven days after booster dose administration, 14 CCGs were significantly elevated compared to the levels measured just before the booster dose administration. Interestingly, similar to alterations observed after administration of the primer dose, upregulated CCGs included molecules responsible for the anti-viral IFN responses (IP-10, IFN-γ, and IFN-γ-inducing IL-18), but also inflammatory marker CRP, Th1 cytokines (TNF-α, IL-1β, IL-6, and IL-1Ra), MIP-1α, and eosinophil and B-cell function promoting factor (IL-5) indicating activation of a wide range of immune markers despite the immunocompromised status of these patients (**Figure 2A** **, right panel**). Remarkably, after a non-significant drop one day after the primer dose administration, the levels of IL-5 gradually increased, especially after the administration of the booster dose at day 28. IL-5 is a major eosinophilic factor, but it was originally identified as a B cell growth and differentiation factor in inducing antibody secretion and class switching (29) and fits well with our data of highly upregulated IL-5 levels after the booster dose. Notably, within the power of our study, none of the studied immunomodulatory and Treg CCGs (i.e., IL-10, IL-2, and IL-2Rα) were altered. Levels of vascular injury marker VCAM-1 and angiogenesis markers BDNF, bFGF and VEGF-A were also upregulated after booster dose administration (day 28), compared to the levels just before administration of the booster dose (day 21) (**Figure 2A**, right panel). Notably, angiogenic markers bFGF, BDNF and VEGF-A were significantly increased at both day 21 and day 28 compared to day 0 (**Figure 2B**). An independent regression analysis also showed that these angiogenic markers along with VEGF receptor (Flt-1) were significantly increasing over time after the primer dose administration (**Supplementary Figure 1**). However, in absence of a non-vaccinated cancer patient group it is difficult to ascertain whether this significant increase in angiogenic markers is the effect of vaccination or a part of natural progression of cancer in these patients.

We previously reported in this cohort that local or systemic adverse events (AEs) were mostly mild to moderate with only 3% (n = 5) and 6% (n =12) patients experiencing severe local or systemic AEs after primer and booster dose, respectively (5). Local reactogenicity was graded as mild, moderate, or severe. Systemic AEs were recorded according to the Common Terminology Criteria for Adverse Events version 5.0 (CTCAE v5.0; graded 0–5; grade 5 being death). Additionally, investigating whether CCG responses are different in patients who developed severe AE, only PlGF was observed to be significantly downregulated after the primer dose in uncorrected paired t-test statistics (*p* = 0.027) and was not significant after *post-hoc* false discovery rate correction. These data fit well with studies suggesting that systemic adverse events noted after vaccination in cancer patients are not necessarily vaccine related (5, 16).

### 3.2 Type of cancer therapy does not majorly alter the CCG profile induced by the BNT162b2 mRNA vaccine

**Figure 3:**
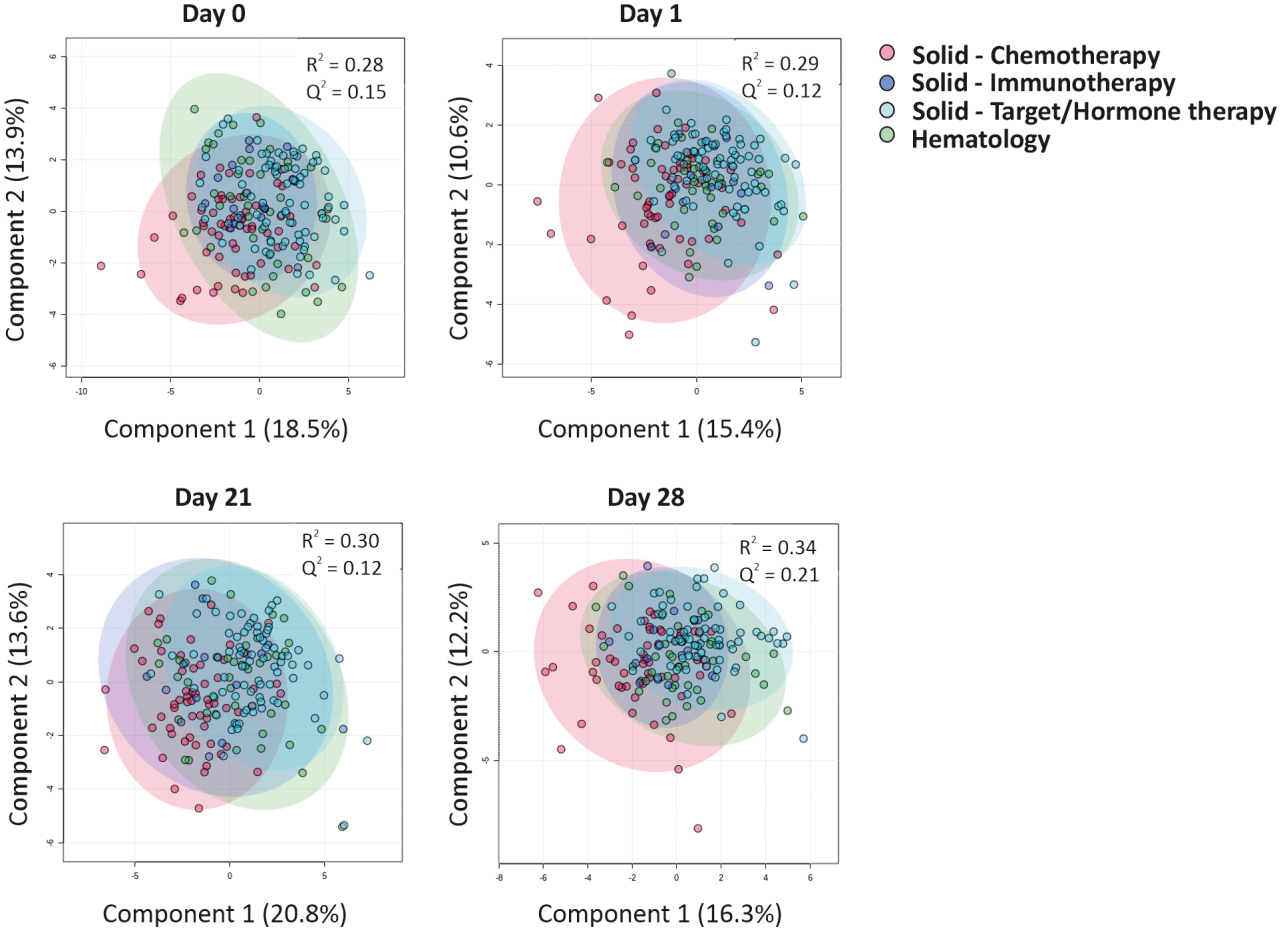
CCGs alterations as a response to primer and booster vaccinations in cancer patients undergoing different treatment regimens. Cluster analyses of CCGs at different timepoints with a Partial Least Squares-Discriminant Analysis (PLS-DA) reveal minor differences between patients undergoing distinct types of anti-cancer therapies. Hematological patients included patients receiving rituximab or patients who received an allogeneic hematopoietic stem cell transplantation at least one year before the primer dose vaccination.

An inadequate IgG immune response to the BNT162b2 mRNA vaccine was reported especially in hematological malignancy patients and notably in those receiving rituximab, an anti-CD20 B cell blocker (5). We thus first questioned whether CCG profiles could discriminate hematological cancer patients receiving rituximab from those receiving stem cell transplantation, the other major treatment modality for hematological malignancy patients studied in this report, or from all other cancer and treatment groups combined. A significant discrimination was observed at day 1 for hematological cancer patients with or without rituximab (accuracy = 87%; R^2^ = 0.67; Q^2^ = 0.30), but was not observed at other timepoints, nor was observed at any timepoint when combining the groups of solid cancer and non-rituximab-treated hematological cancer patients (**Supplementary Figure 2**). As other treatment modalities, especially chemotherapy for patients with solid tumors, also showed a diminished immune response, we performed a similar discriminant analysis that showed no significant underlying difference in CCG profiles at any timepoint (**Figure 3** **;** **Supplementary Figure 3**).

Studying individual cytokines in a difference of mean analysis revealed that the only cytokine linked to vaccine administration and upregulated in all treatment groups was IP-10, which is indicative of an effective anti-viral immune response. Moreover, IP-10-regulator IFN-γ was also upregulated in patients with solid tumors treated with targeted or hormonal therapy (**Supplementary Figure 3**). Surprisingly, neutrophil chemoattractant IL-8 was downregulated in both hematological malignancy patients and patients with solid tumors treated with targeted/hormonal therapy. Moreover, all groups of patients with solid tumors demonstrated a significant downregulation of IL-17A, a pro-inflammatory cytokine involved mainly in the activation of neutrophils. Lastly, the solid tumor cohort treated with chemotherapy or targeted/hormone therapy showed a significant increase of VEGF-C, bFGF, and BDNF over 21 days (**Supplementary Figure 3**). These data indicate that except for rituximab-treated hematological malignancy groups that behave differently at day 1, type of cancer therapy is not a major driver for the observed CCG profiles induced by the BNT162b2 vaccination.

### 3.3 CRP, IL-15, IL-18, and PlGF predict a poor BNT162b2 immune response in cancer patients

**Figure 4:**
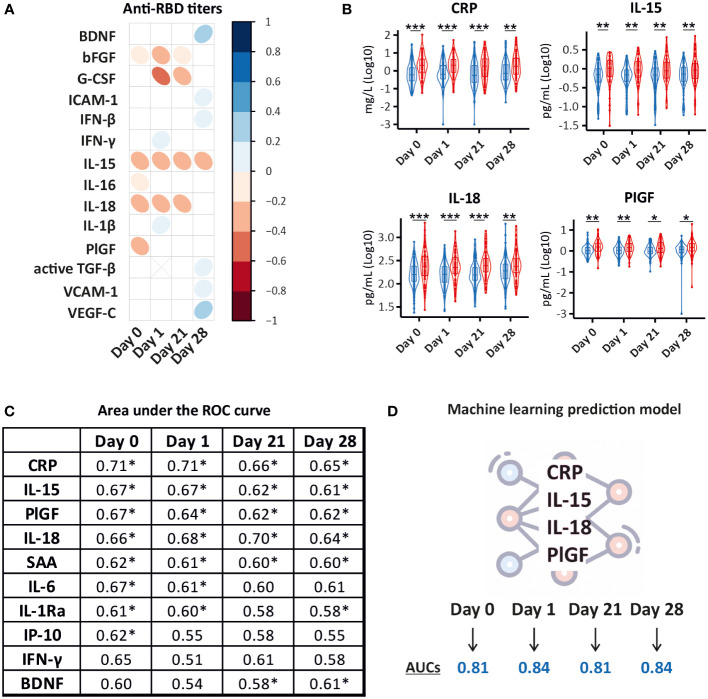
Prediction models for BNT162b2 immune response in cancer patients. **(A)** Correlation matrix depicting the correlation between CCG measurements (log_10_ transformed) and quantitative anti-RBD IgG measurements at day 49. IgG antibody levels to SARS-CoV-2 RBD antigen were assessed with an enzyme-linked immunosorbent assay for quantitative detection of IgG antibody levels to SARS-CoV-2 RBD antigen. Only CCGs with significant correlations are shown. **(B)** Significantly different between good (≥ 200 IU/mL) (blue) and poor (< 200 IU/mL) (red) responders to the BNT162b2 vaccine. A good/poor responder threshold of anti-RBD IgG titer of 200 IU/mL used in this study predicts a neutralization response required for 50% protection against symptomatic SARS-CoV-2 infection (99%-100% specificity at a sensitivity of 95%). *p < 0.05, **p < 0.01, ***p < 0.001. **(C)** Area Under the Receiver Operating Characteristic (AUROC) values for 10 predictors of the binary IgG response as good or poor responders at day 0, day 1, day 21, and day 28. * Denotes significant *p*-values of at least < 0.05. **(D)** Random Forest Classifier predicted a model where a combination of CRP, IL-15, IL-18, and PlGF levels measured right before vaccine administration (day 0) and at day 1, day 21, and day 28 after the primer dose predicted good and poor responders with high accuracy (AUCs depicts averages of 10 individually constructed ROC curves).

Due to the limited ability of some patients with solid or hematological malignancies to develop a protective antibody response, we aimed to identify a unique CCG signature in cancer patients that could differentiate good from poor responders to BNT162b2 vaccination. For this, we examined the relationship between alterations in the studied CCGs at all sampling timepoints (day 0, day 1, day 21, and day 28) with levels of anti-RBD titers measured 28 days after the administration of the booster dose of the BNT162b2 vaccine (day 49) (**Figure 1**). This was done following several approaches. First, we utilized anti-RBD titers measured at day 49 as a continuous variable and correlated with CCGs at all studied timepoints. Amongst others, BDNF, VEGF-C, IFN-γ, IFN-β, and ICAM-1 were significantly positively associated with anti-RBD titers at one or several timepoints (**Figure 4A**). Additionally, bFGF, PIGF, IL-18, G-CSF, and pro-inflammatory cytokines IL-15 and IL-16 were significantly negatively associated with anti-RBD titers (**Figure 4A**). These data suggest that pre-existing and sustained CCG signatures in patients with solid and hematological malignancies can be predictive of the quantitative antibody response post-BNT162b2 vaccination.

Since the primary outcome of this study was the assessment of the level of protection conferred by vaccination, we further utilized a threshold of 200 IU/mL shown to predict a neutralization response conferring 50% protection against SARS-CoV-2 infection in our prior study (5). Examining the ability of CCGs to predict poor responders (< 200 IU/mL) from good responders (≥ 200 IU/mL), 4 CCGs were identified to be significantly different at all studied timepoints that included CRP, IL-15, IL-18, and PlGF (**Figure 4B**).

Area under the curve receiver operating characteristic (AUROC) analysis was further performed to discriminate between good and poor responders. AUROC was constructed for each CCG and the top discriminant CCGs were utilized to build models. Performance was studied for each timepoint to assess the capability of the model to sustain at all studied timepoints. While inflammatory marker CRP on its own did not emerge as a good classifier (see **Supplementary Table 2** for more details) and did not correlate with anti-S or anti-RBD antibody titers measured as a continuous variable (**Figure 4A**), a highly significant difference was observed in CRP levels in good and poor responders at all studied timepoints (**Figure 4B**). Moreover, prior to vaccine administration, the upregulated inflammatory marker CRP showed the highest predictive value for vaccine response followed by NK-cell inducer IL-15, PlGF, IL-6, IL-18, and serum amyloid A (SAA). One day after administration of the primer dose, CRP, IL-18, IL-15, PlGF, IL6, and SAA remained in the signature predicting a worse qualitative antibody response. Similarly, prior to administration of the booster dose, the signature included CRP, IL-15, IL-18, PlGF, and SAA (**Figure 4C**).

Lastly, we performed Random Forest classification that validated the signature consisting of CRP, IL-15, IL-18, and PlGF, differentiating good from poor anti-SARS-CoV-2 BNT162b2 vaccine responders with more than 80% accuracy. Interestingly, this signature was maintained until day 28 after the administration of the primer dose (**Figure 4D**; **Supplementary Figure 4**).

## 4 Discussion

In this study, we show an alteration of a diverse group of inflammatory mediators and growth factors that includes interferons, Th1, Th2, and Th17 cytokines, as well as some markers of angiogenesis and vascular injury in a heterogeneous population of patients with solid or hematological malignancies vaccinated with BNT162b2. In a previous study, some of these CCGs including IFN-γ, IP-10, TNF-α, IL-6, IL-1Ra, CRP, MIP-1α, and VEGF-A were shown to be upregulated upon BNT162b2 administration in healthy individuals (12); however, the upregulation noted in healthy volunteers (up to 20-fold) is substantially higher than noted in our population of cancer patients (up to 2-fold). Additionally, an increase in IFN-γ and IP-10 levels was also observed in an elderly population upon administration of the BNT162b2 vaccine but at a larger magnitude than detected in our cohort (17). These data suggest that BNT162b2 vaccine administration in cancer patients can generally elicit an anti-SARS-CoV-2-driven immune response that is similar in pattern, but not in magnitude, to healthy individuals. Even though our study is restricted to the BNT162b2 vaccine, we expect to observe similar alterations upon administration of other mRNA COVID-19 vaccines as well as non-mRNA vaccines, although they show a more pronounced upregulation of pro-inflammatory responses at least after the administration of the primer dose (30).

All cohorts of patients with solid and hematological malignancies undergoing different treatment regimens developed anti-viral interferon responses after vaccination with BNT162b2. However, with the exception of the rituximab treatment cohort, no major underlying differences in CCG profiles were identified between different cancer or treatment groups at any timepoint. These data suggest that despite having different tumor types and undergoing different therapies, patients respond similarly to vaccination with BNT162b2.

Previous studies have shown that antibody titers in patients with certain cancers, including but not limited to, advanced cancers and B cell hematological malignancies, are either absent or very low not only after SARS-CoV-2 infection, but also after SARS-CoV-2 vaccination (5, 7–11, 31–34). In line with the major aim of this study, we identified a unique immune signature based on upregulated CRP, IL-15, IL-18, and PlGF that could be used to identify patients who did not sufficiently respond to vaccination with BNT162b2 vaccine. The signature was present at different studied timepoints before or after vaccination and was not majorly affected by different anti-cancer treatments. We believe that this unique biomarker signature would not only be useful for clinicians in identifying cancer patients at increased risk of developing SARS-CoV-2 for better patient care, but also be able to guide health policies in categorizing cancer patients in need of enhancer vaccine doses or pre-exposure prophylaxis with synthetic monoclonal antibodies to protect potential non-responders to the BNT162b2 vaccine.

Lastly, our data also suggest that pro-inflammatory cytokines and growth factors interact to dictate an inherent immune response in cancer patients that could generally render them refractive to other immune interventions. Whether the identified signature or similar immune-based CCG profiles can be predictive of primary resistance to immunotherapy, observed in approximately 12% of the patients (35), remains open to future investigations.

## Data availability statement

The raw data supporting the conclusions of this article will be made available by the authors, without undue reservation.

## Ethics statement

The studies involving human participants were reviewed and approved by Antwerp University Hospital. The patients/participants provided their written informed consent to participate in this study.

## Author contributions

Conceptualization: SK-S, PD, MP; Funding acquisition: SK-S, PD, MP, ET; Overall study supervision: SK-S; Clinical data and sample collection: LV, L-AT, GV, SR, IM, SK, YD, GM, BP; Biobanking: MH; CCG analysis: AK, FW, AG, AH; Serology and seroneutralization analyses: PP, KN, KA, MB, ID, MG; Statistical analysis: AK, FW, AG, AH; Data curation: AK, AG, ER; Data interpretation: SK-S, PD, MP, TV, SM-K, AK, FW, AG, AH, MB; Manuscript writing: SK-S, AK, FW, AG, PD, AH, MB; All authors read, gave input, and approved the final manuscript.
