# Supplementary material for: Predictive model for BNT162b2 vaccine response in cancer patients based on blood cytokines and growth factors
Source: Front Immunol. 2022 Dec 22;13:1062136. doi: 10.3389/fimmu.2022.1062136 (PMC9813584; doi:10.3389/fimmu.2022.1062136)
Supplement: Supplementary file 1 [file DataSheet_1.docx]

Supplementary Material

# Supplementary Methods

## Sample collection and processing

Whole blood was prospectively collected in heparin blood and serum collection tubes. Within 3 hours of blood collection, plasma was prepared by centrifuging twice at 1900 × g for 10 minutes without brakes. Blood in the serum collection tubes was allowed to clot thoroughly for 60 minutes and serum was prepared by centrifuging at 1300 × g for 10 minutes without brakes. Aliquots were flash frozen in liquid nitrogen and stored in the Biobank of Antwerp University Hospital at -80°C until further analysis.

## Cytokine, chemokine and growth factor (CCG) measurements in plasma

CCGs were measured in plasma samples on a multiplex platform (Meso Scale Discovery (MSD), MD, USA) using off-the-shelf (V-plex) and customized (U-plex) panels, following manufacturer instructions. Briefly, 96-well plates of the U-plex panels were coated with a capturing antibody coupled to a linker for one hour. The vascular injury panel (K15198D) was washed before use. The angiogenesis panel (K15190D) was first blocked with blocking buffer for one hour. Thereafter, all plates were washed three times with PBS-Tween (0.05%). Samples were incubated for one hour (except for the angiogenesis and the vascular injury panels, where two hours of incubation were performed), after which the plates were washed another three times. Detection antibody with a SULFO-TAG was added and after another one-hour incubation step (two hours for the angiogenesis panel), the plates were washed and read with MSD reading buffer on the QuickPlex SQ 120 (MSD) as previously described [1].

# Supplementary Figures and Tables


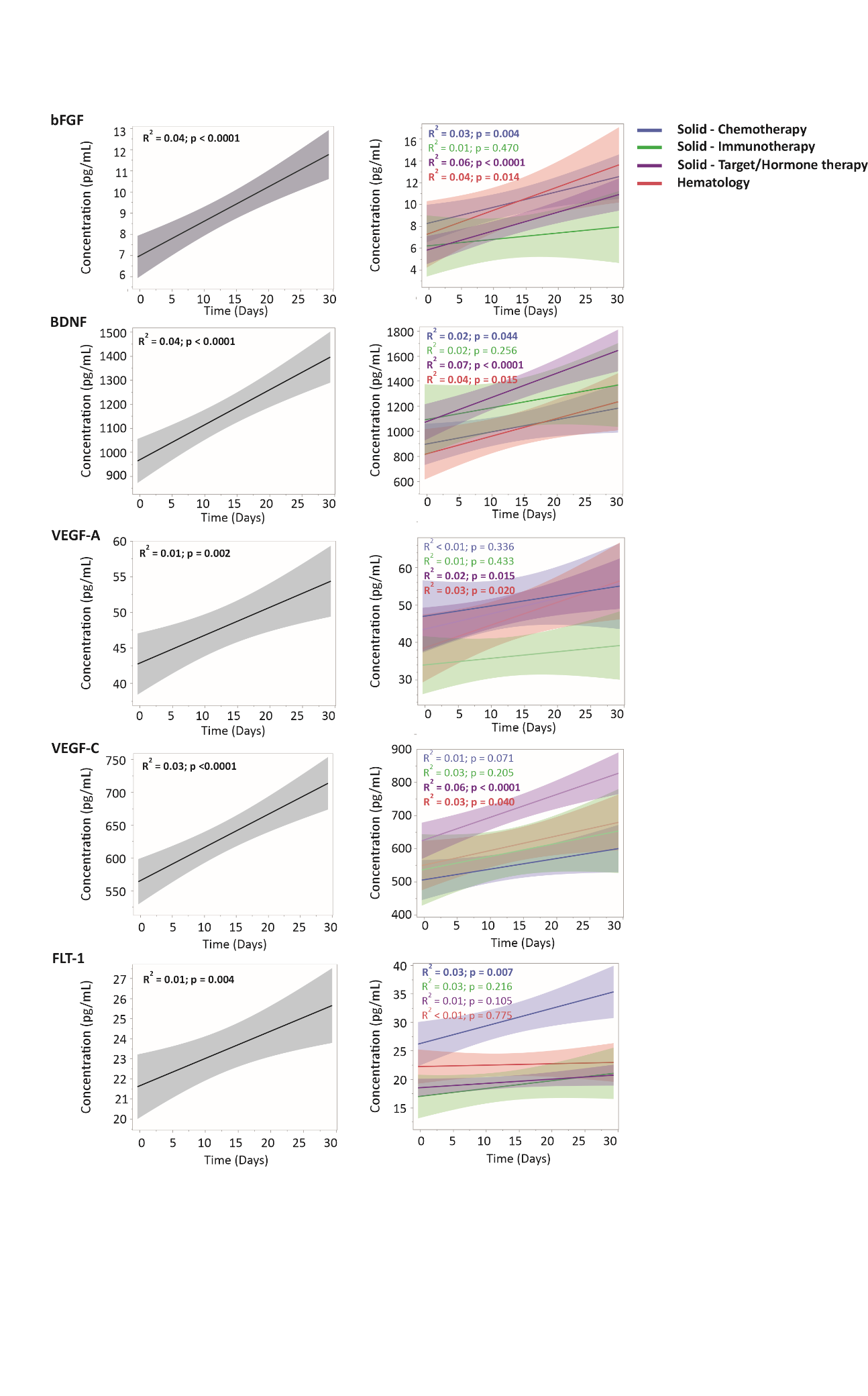


Supplementary Figure 1. Temporal alterations in cytokines, chemokines and growth factors (CCGs) levels in vaccinated cancer patients. Time is represented as days since primer dose vaccination. *P*-values in the graph refer to significance of the slope of the regression lines.


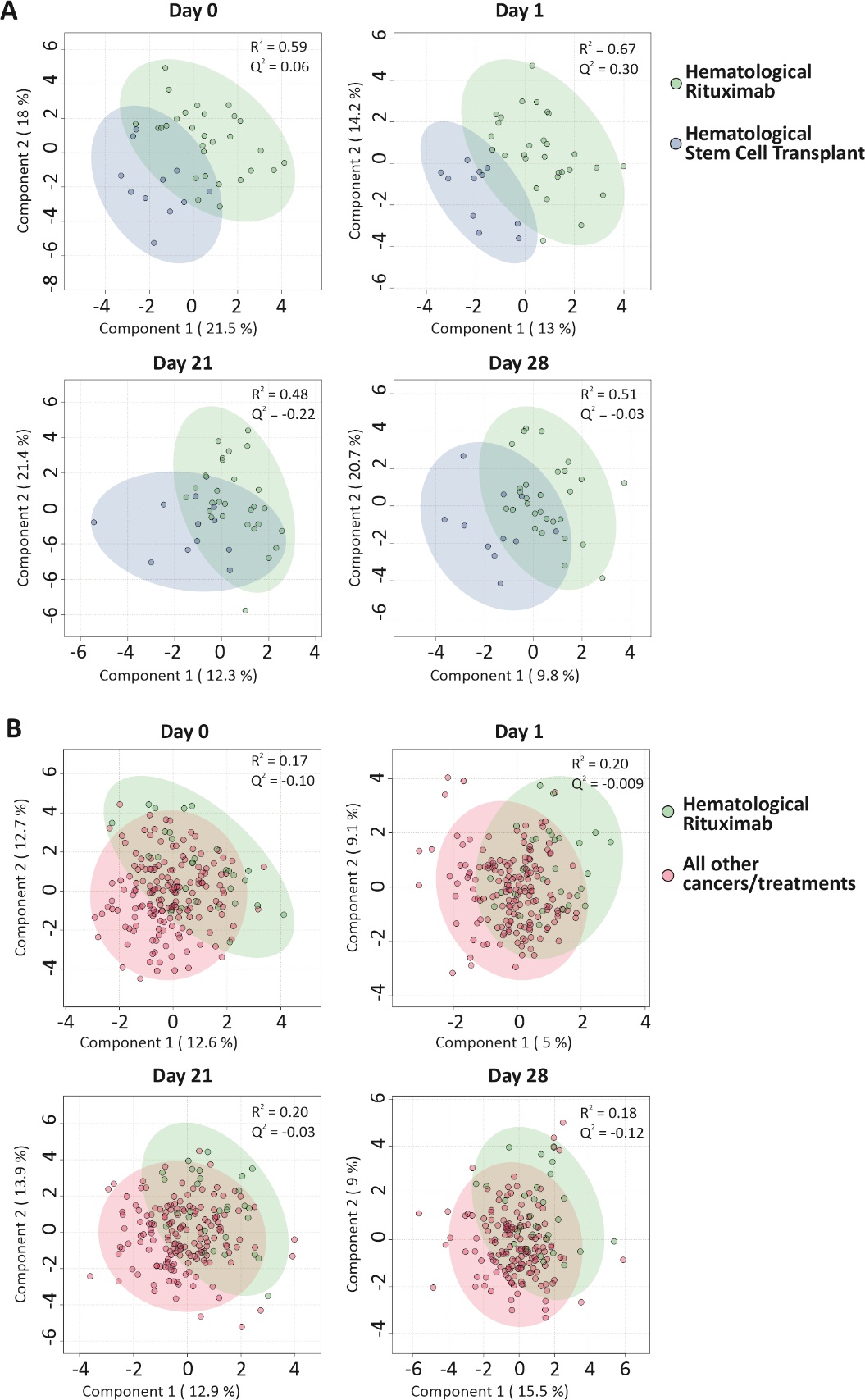


**Supplementary Figure 2**. **CCG analysis in patients with and without hematological malignancies.** Cluster analyses of CCGs at different timepoints with partial least squares-discriminant analysis (PLS-DA) reveal (**A**) differences between patients with hematological malignancies treated with rituximab and patients that received a stem cell transplantation, or (**B**) between patients with hematological malignancies treated with rituximab versus all other cancers/treatments group


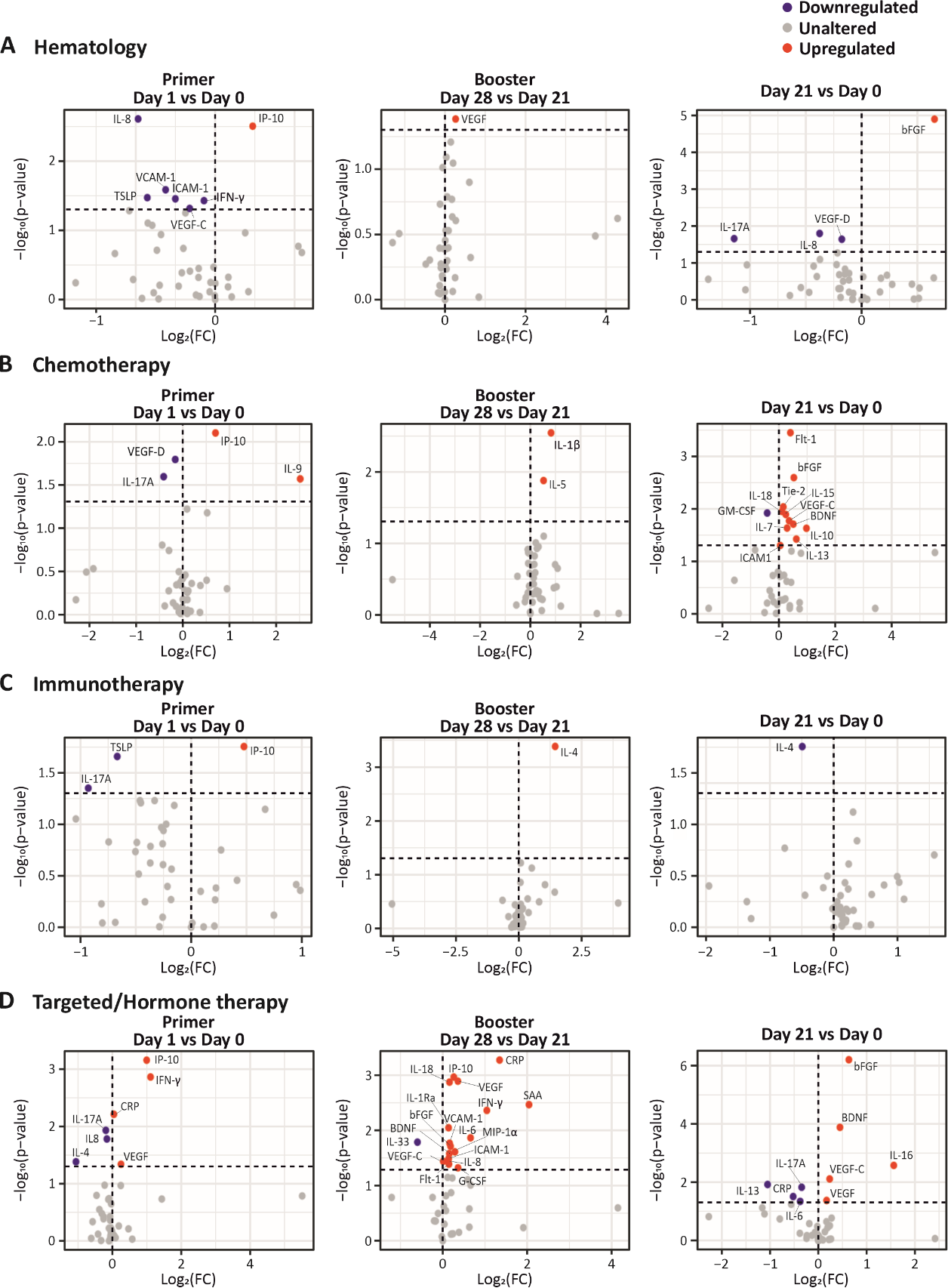


**Supplementary Figure 3**. Volcano plots depicting differentially expressed CCGs after the administration of the primer and booster doses compared to the CCG levels prior to vaccine administration (**A**) in cancer with hematological malignancies, (**B**) patients with solid cancers treated with chemotherapy, (**C**) immunotherapy and (**D**) targeted or hormonal therapy. *P*-values were calculated using paired t-test. The vertical dotted line represents no change. The horizontal dotted line represents a *p*-value of 0.05.


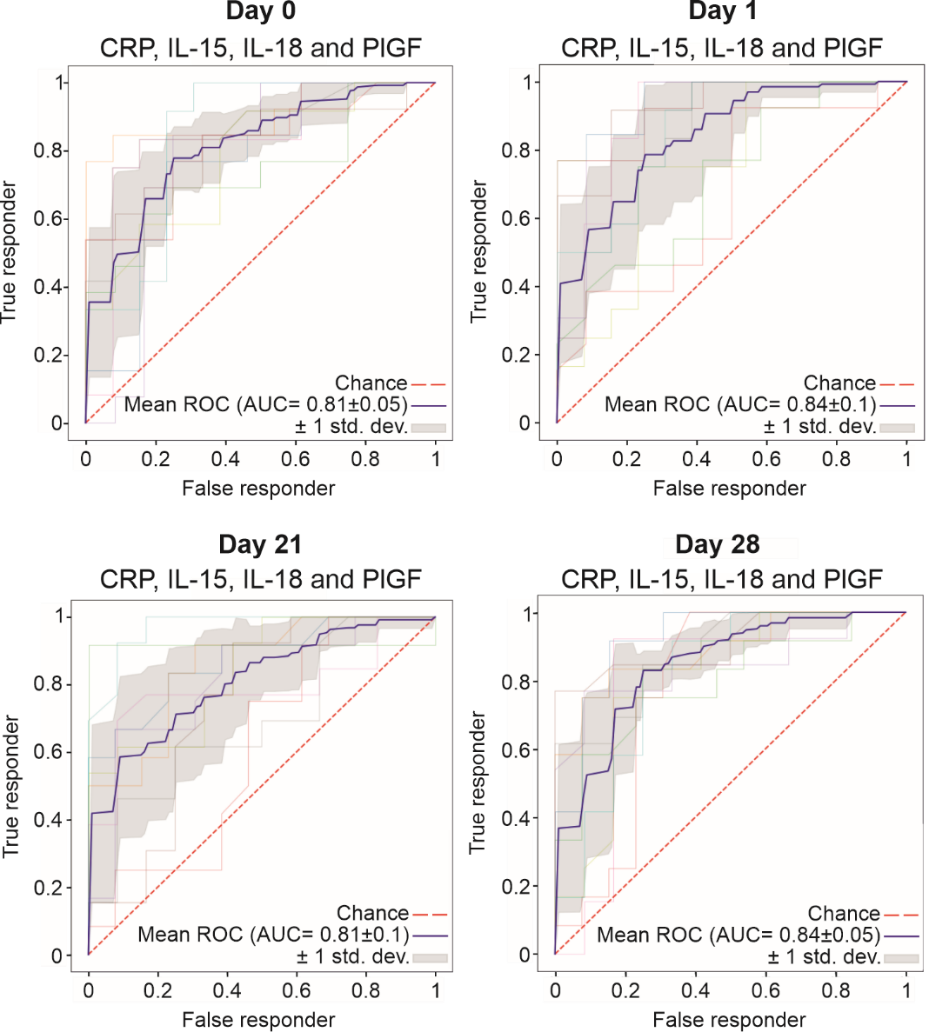


**Supplementary Figure 4**. ROC curves for the combination of CRP, IL-15, IL-18 and PlGF in a random forest classifier model with Synthetic Minority Oversampling Technique (SMOTE) for the prediction of the qualitative IgG response (good versus poor responder) are depicted for day 0, day 1, day 21 and day 28.

| **Supplementary Table 1**. **Patient characteristics**  *Percentage of total patients with solid tumors. | | | | | |
| --- | --- | --- | --- | --- | --- |
| **Demographics** | **Target/hormone therapy  (*n* = 79)** | **Immuno-therapy**  **(*n* = 16)** | **Chemo-therapy  (n = 63)** | **Hema-tological  (*n* = 41)** | **Overall  (n = 199)** |
| Sex, *n* (%) | | | | | |
| Female | 70 (88.6) | 4 (25.0) | 43 (68.3) | 17 (41.5) | 134 (67.3) |
| Male | 9 (11.4) | 12 (75.0) | 20 (31.7) | 24 (58.5) | 65 (32.7) |
| Age, years | | | | | |
| Mean (SD) | 59.5 (12.1) | 68.3 (8.09) | 60.0 (13.2) | 61.2 (11.5) | 60.7 (12.2) |
| Median (range) | 60.0 (31.0-86.0) | 69.5 (56.0-84.0) | 61.0 (26.0-88.0) | 63.0 (25.0-79.0) | 62.0 (25.0-88.0) |
| BMI | | | | | |
| Mean (SD) | 25.7 (4.74) | 27.0 (4.13) | 25.5 (5.19) | 25.2 (3.88) | 25.6 (4.67) |
| Median (range) | 25.5 (17.8-40.0) | 26.9 (19.7-34.5) | 24 (18.9-44.8) | 24.4 (17.1-35.5) | 25.1 (17.1-44.8) |
| Missing, *n* (%) | 0 (0) | 0 (0) | 3 (4.8) | 2 (4.9) | 5 (2.5) |
| ECOG score, *n* (%) | | | | | |
| 0 | 73 (92.4) | 11 (68.8) | 48 (76.2) | 38 (92.7) | 170 (85.4) |
| 1 | 6 (7.6) | 5 (31.2) | 13 (20.6) | 3 (7.3) | 27 (13.6) |
| 2 | 0 (0) | 0 (0) | 1 (1.6) | 0 (0) | 1 (0.5) |
| Missing | 0 (0) | 0 (0) | 1 (1.6) | 0 (0) | 1 (0.5) |
| Autoimmune disease, *n* (%) | 4 (5.1) | 0 (0) | 1 (1.6) | 3 (7.3) | 8 (4.0) |
| Kidney disease, *n* (%) | 1 (1.3) | 1 (6.2) | 5 (7.9) | 1 (2.4) | 8 (4.0) |
| Hypertension,  *n* (%) | 20 (25.3) | 4 (25.0) | 22 (34.9) | 8 (19.5) | 54 (27.1) |
| Diabetes, *n* (%) | 3 (3.8) | 2 (12.5) | 10 (15.9) | 5 (12.2) | 20 (10.1) |
| Coronary disease, *n* (%) | 4 (5.1) | 2 (12.5) | 10 (15.9) | 7 (17.1) | 23 (11.6) |
| Smoking status, *n* (%) | | | | | |
| Current smoker | 5 (6.3) | 1 (6.2) | 5 (7.9) | 2 (4.9) | 13 (6.5) |
| Former smoker | 21 (26.6) | 11 (68.8) | 21 (33.3) | 18 (43.9) | 71 (35.7) |
| Non-smoker | 51 (64.6) | 3 (18.8) | 29 (46.0) | 21 (51.2) | 104 (52.3) |
| Missing | 3 (2.5) | 1 (6.2) | 8 (12.7) | 0 (0) | 11 (5.5) |
| Stage, *n* (%) | | | | | |
| I | 20 (25.3) | 0 (0) | 6 (9.5) | NA | 26 (16.5)* |
| II | 19 (24.1) | 2 (12.5) | 6 (9.5) | NA | 27 (17.1)* |
| III | 6 (6.3) | 2 (12.5) | 6 (9.5) | NA | 14 (8.2)* |
| IV | 33 (41.8) | 12 (75.0) | 42 (66.7) | NA | 87 (55.1)* |
| Missing | 2 (2.5) | 0 (0) | 3 (4.8) | NA | 46 (29.1)* |

| **Supplementary Table 2**. **Predictive value of CRP with outcome good responder versus poor responder.** Although CRP had an AUC of 0.71, at the clinical cut-off of 4 mg/L it had only a sensitivity of 30% and a specificity of 88% at baseline day 0. If used to identify patients that would benefit from adjuvant therapy, too many patients would be missed. An optimal cut-off of CRP to differentiate poor from good responders was 1 mg/L that provided a sensitivity and specificity of 72% and 61%, respectively. | | | | |
| --- | --- | --- | --- | --- |
| Cut-Off | Sensitivity | Specificity | Positive likelihood ratio | Negative Likelihood ratio |
| 1 mg/L | 72% | 61% | 1.85 | 0.46 |
| 2 mg/L | 49% | 81% | 2.59 | 0.63 |
| 4 mg/L | 30% | 88% | 2.48 | 0.80 |
| 10 mg/L | 13% | 96% | 3.28 | 0.91 |

References

1. De Winter FHR, Hotterbeekx A, Huizing MT, Konnova A, Fransen E, Jongers B, et al. Blood Cytokine Analysis Suggests That SARS-CoV-2 Infection Results in a Sustained Tumour Promoting Environment in Cancer Patients. Cancers (Basel). 2021;13(22). <https://doi.org:10.3390/cancers13225718>
